# Evaluation of robustness in hybrid intensity-modulated radiation therapy plans generated by commercial software for automated breast planning

**DOI:** 10.1038/s41598-022-05538-8

**Published:** 2022-01-26

**Authors:** Norifumi Mizuno, Ryouhei Yamauchi, Jiro Kawamori, Tomoko Itazawa, Munefumi Shimbo, Keiichiro Nishimura, Takafumi Yamano, Shogo Hatanaka, Masatsugu Hariu, Takeo Takahashi

**Affiliations:** 1 Department of Radiation Oncology, St. Luke’s International Hospital, 9-1 Akashi-cho, Chuo-ku, Tokyo, 104-8560 Japan; 2 Department of Radiation Oncology, Saitama Medical Center, Saitama Medical University, 1981 Kamoda, Kawagoe, Saitama 350-8550 Japan

**Keywords:** Radiotherapy, Breast cancer

## Abstract

This study aimed to evaluate the robustness against geometric uncertainties in the hybrid intensity-modulated radiation therapy (IMRT) plans generated by commercially available software for automated breast planning (ABP). The ABP plans were compared with commonly used forward-planned field-in-field (FIF) technique plans. The planning computed tomography datasets of 20 patients who received left-sided breast-conserving surgery were used for both the ABP and FIF plans. Geometric uncertainties were simulated by shifting beam isocenters by 2, 3, 5, and 10 mm in the six directions: anterior/posterior, left/right, and superior/inferior. A total of 500 plans (20 patients and 25 scenarios, including the original plan) were created for each of the ABP and FIF plans. The homogeneity index of the target volume in the ABP plans was significantly better (*p* < 0.001) than the value in the FIF plans in the scenarios of shifting beam isocenters by 2, 3, and 5 mm. Mean heart dose and percentage volume of lungs receiving a dose more than 20 Gy were clinically acceptable in all scenarios. The hybrid IMRT plans generated by commercially available ABP software provided better robustness against geometric uncertainties than forward-planned FIF plans.

## Introduction

Breast cancer is the most common cancer affecting women in many countries, with more than 2 million breast cancer patients per year worldwide^1^. Breast conservation therapy is the standardized treatment for early-stage breast cancer, and whole breast irradiation after breast-conserving surgery has been established for reducing local recurrence and breast cancer mortality^2–5^. Several advanced techniques such as intensity-modulated radiation therapy (IMRT) have been developed in the past 20 years. The tangential breast IMRT technique has been applied to improve target dose homogeneity and reduce excessive high-dose regions (called “hot spots”) for whole breast irradiation. Several randomized controlled trials indicated that breast IMRT reduces acute toxicities, such as edema, erythema, moist desquamation, and breast pain, compared with the conventional physical wedge technique^6–9^. Generally, more expertise and planning time are required to generate a breast IMRT plan than to create a plan with the conventional physical wedge technique. Therefore, automated breast planning (ABP) software has been developed to decrease the cost of planning time in several institutions using in-house programs or commercially available software^10–13^.

One of the disadvantages of the breast IMRT technique is unexpected dose deviations caused by geometric uncertainties in the patient setup and/or respiratory motion^14–17^. One approach to mitigate the effect of geometric uncertainties is the use of a hybrid technique. A hybrid IMRT plan includes not only inverse-planned IMRT fields but also glancing open fields for breast flash. Several previous studies mentioned that the hybrid IMRT plan may have more robustness against geometric uncertainties than a full IMRT plan. However, these investigations were performed in a limited number of scenarios^14,17^. Moreover, hybrid IMRT planning generated by commercially available software for ABP has not yet been investigated.

This study aimed to evaluate the robustness against geometric uncertainties in the hybrid IMRT plans generated by commercially available ABP software. The ABP approach was compared with clinical treatment plans that comprised forward-planned field-in-field (FIF) technique in terms of the target dose and the dose for the organ at risk.

## Methods

### Ethical approval and informed consent

All procedures in studies involving human participants were performed in accordance with the ethical standards of the institutional review board of the St. Luke’s International Hospital and with the 1964 Helsinki Declaration and its later amendments or comparable ethical standards. The study protocol was approved by the institutional review board of the St. Luke’s International Hospital (approval number: 16-R070). Informed consent was obtained from all the patients before the computed tomography (CT) simulation.

### Patient population and CT simulation

A total of 20 patients who received left-sided breast conservation therapy at our institution from September 2016 to August 2017 were prospectively enrolled. The patients received whole breast irradiation after breast-conserving surgery with no regional lymph node irradiation. The CT simulation was performed with a patient lying supine with wing support immobilization (Engineering System Co., Matsumoto, Nagano, Japan), with both arms raised above the head. A LightSpeed RT16 helical CT scanner (GE Healthcare, Waukesha, WI, USA) was used to acquire images at a slice thickness of 2.5 mm without breath holding.

### Treatment instrument and prescribed dose

A Clinac 21EX linear accelerator (Varian Medical Systems, Palo Alto, CA, USA) with an 80-leaf Millennium multileaf collimator was used as the treatment machine. A total dose of 42.56 Gy in 16 fractions was used for the prescribed dose. All treatment plans were composed with 4-MV photon beams, and collapsed cone convolution superposition was selected for the dose calculation algorithm. The grid size for dose calculation was set to a constant value of 2 mm.

### FIF plans

The Pinnacle^3^ radiation treatment system (version 9.10, Philips Radiation Oncology Systems, Fitchburg, WI, USA) was used for clinical treatment planning. Forward-planned FIF plans that comprised two tangential open beams and 1–3 subfields at the same gantry angles were generated. The mammary gland as the clinical target volume (CTV), heart, and lungs were defined in accordance with the European Society for Radiotherapy and Oncology (ESTRO) consensus guidelines. The planning target volume was defined as the CTV plus a three-dimensional 10-mm margin (posterior side: 5 mm). The heart and lungs were shielded by a multileaf collimator for the dose reduction. A point-dose prescription for the reference point within the CTV was performed. The details of the FIF plans in our institution have been described in previous studies^13,18^.

### ABP plans

The ABP plans were generated by RayStation software (version 4.7.4.4; RaySearch Laboratories, Stockholm, Sweden, the algorithm of ABP has been confirmed to be the same from version 4 to 10)^10,11,19^. Whole breast site and breast coverage modes from the default planning parameters in the ABP software settings were applied. The tangential breast plan in this ABP software is hybrid IMRT, comprising two opposed open fields and the inversely optimized IMRT fields set at the same gantry angles. The open fields were weighted to approximately 80% of the total monitor units (MUs)^10,13^. The average dose prescription was performed for an automatically defined CTV as a normal setting of the ABP software. Our previous study detailed the characteristics of the ABP plan and the clinical acceptability^13^.

### Simulations of setup errors and dose-volume data comparison

Setup errors were simulated by shifting beam isocenters by 2, 3, 5 and 10 mm in the six directions: anterior/posterior, left/right, and superior/inferior. A total of 500 plans (20 patients and 25 scenarios, including the original isocenters plan) were created for each of FIF plans and ABP plans. The influence of the shift of isocenters was assessed by the target dose region of original isocenters plans, heart, and lungs. The prescribed 90% dose volume was created as the target dose region from the isodose line in each of the FIF and ABP original isocenters plans. Manually defined heart and lungs were used in this evaluation as the gold standard, although ABP software automatically generates the structures. Dose-volume data regarding the homogeneity index (*HI*) of 90% dose volume, mean dose (*D*_mean_) of the heart, and percentage volume of bilateral lungs receiving dose greater than 20 Gy (*V*_20 Gy_) were recorded. *HI* was defined from the International Commission on Radiation Units and Measurements (ICRU) Report 83 as follows^20^:

1 $$HI = \left( {D_{{{2}\% }} {-}D_{{{98}\% }} } \right)/D_{{{5}0\% }}$$

where *D*_x%_ is the absorbed dose received by *x*% of the volume.

### Statistical analysis

We used R software (version 3.4.1, R Foundation for Statistical Computing, Vienna, Austria) for statistical analysis^21^. A paired *t-*test was used for comparisons between FIF plans and ABP plans, with *p* < 0.05 considered significant.

## Results

### Target dose and homogeneity

**Figure 1:**
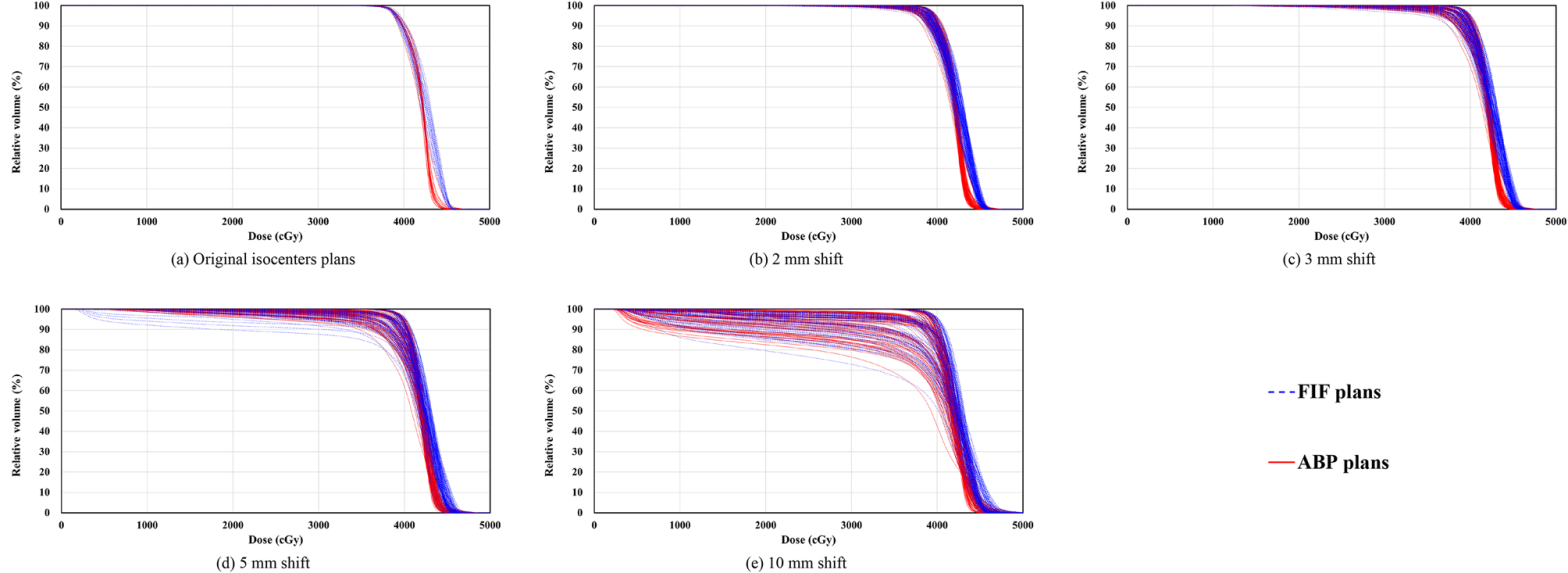
Comparison of dose-volume histograms of 90% dose-volume among FIF plans and ABP plans. FIF, field-in-field technique; ABP, automated breast planning. The prescribed 90% dose volume was created from isodose line in each FIF and ABP original isocenters plans.

Table 1 presents the analysis of dose-volume data for the target dose and homogeneity using 90% dose volume of original isocenters plans as the target dose region. The mean *D*_98%_ values of FIF and ABP plans at the original isocenters were 3843 ± 18 cGy and 3843 ± 12 cGy, respectively (*p* = 0.958). The *D*_2%_ and *HI* values of the original ABP plans (4428 ± 48 cGy and 0.138 ± 0.011) were significantly lower (*p* < 0.001) than the value in the original FIF plans (4526 ± 14 cGy and 0.160 ± 0.005). In the scenarios of shifting beam isocenters by 2, 3, 5, and 10 mm, the *D*_2%_ and *HI* values of ABP plans (*D*_2%_: 4435 ± 46 cGy, 4441 ± 47 cGy, 4461 ± 51 cGy, 4523 ± 74 cGy; *HI*: 0.151 ± 0.023, 0.173 ± 0.052, 0.282 ± 0.185, 0.549 ± 0.351) were significantly lower (*p* < 0.001) than the value in FIF plans (*D*_2%_: 4530 ± 21 cGy, 4534 ± 27 cGy, 4545 ± 39 cGy, 4581 ± 65 cGy; *HI*: 0.174 ± 0.028, 0.197 ± 0.063, 0.297 ± 0.178, 0.565 ± 0.330), except for *HI* of 10-mm shifted plans (*p* = 0.086). Figure 1 shows the dose-volume histograms of 90% dose volume among FIF plans and ABP plans. The ABP plans exhibited better target dose coverage and homogeneity than the FIF plans in the scenarios of shifting beam isocenters by 2, 3 and 5 mm.

**Table 1:** Analysis of dose-volume data of 90% dose volume.

Isocenter shift (mm)	*D*_98%_ (cGy)		*p*	*D*_2%_ (cGy)		*p*	*HI*		*p*
	FIF plans	ABP plans		FIF plans	ABP plans		FIF plans	ABP plans	
0	3843 ± 18 [3809–3890]	3843 ± 12 [3816–3866]	0.958	4526 ± 14 [4503–4543]	4428 ± 48 [4372–4553]	< 0.001	0.160 ± 0.005 [0.152–0.172]	0.138 ± 0.011 [0.121–0.167]	< 0.001
	(90.3 ± 0.4% [89.5–91.4])	(90.3 ± 0.3% [89.7–90.8])		(106.4 ± 0.3% [105.8–106.8])	(104.0 ± 1.1% [102.7–107.0])				
2	3792 ± 102 [3324–3923]	3797 ± 93 [3508–3917]	0.161	4530 ± 21 [4485–4576]	4435 ± 46 [4365–4573]	< 0.001	0.174 ± 0.028 [0.134–0.287]	0.151 ± 0.023 [0.114–0.214]	< 0.001
	(89.1 ± 2.4% [78.1–92.2])	(89.2 ± 2.2% [82.4–92.0])		(106.4 ± 0.5% [105.4–107.5])	(104.2 ± 1.1% [102.6–107.4])				
3	3695 ± 245 [2570–3931]	3710 ± 217 [2918–3924]	0.083	4534 ± 27 [4475–4599]	4441 ± 47 [4363–4585]	< 0.001	0.197 ± 0.063 [0.132–0.471]	0.173 ± 0.052 [0.114–0.365]	< 0.001
	(86.8 ± 5.8% [60.4–92.4])	(87.2 ± 5.1% [68.6–92.2])		(106.5 ± 0.6% [105.1–108.1])	(104.3 ± 1.1% [102.5–107.7])				
5	3286 ± 717 [1167–3941]	3277 ± 773 [1191–3933]	0.711	4545 ± 39 [4454–4644]	4461 ± 51 [4362–4615]	< 0.001	0.297 ± 0.178 [0.130–0.817]	0.282 ± 0.185 [0.119–0.810]	< 0.001
	(77.2 ± 16.8% [27.4–92.6])	(77.0 ± 18.2% [28.0–92.4])		(106.8 ± 0.9% [104.7–109.1])	(104.8 ± 1.2% [102.5–108.4])				
10	2201 ± 1333 [354–3932]	2229 ± 1448 [271–3928]	0.446	4581 ± 65 [4458–4735]	4523 ± 74 [4373–4715]	< 0.001	0.565 ± 0.330 [0.128–1.049]	0.549 ± 0.351 [0.139–1.116]	0.086
	(51.7 ± 31.3% [8.3–92.4])	(52.4 ± 34.0% [6.4–92.3])		(107.6 ± 1.5% [104.7–111.3])	(106.3 ± 1.7% [102.7–110.8])				

*D*_*x*%_, absorbed dose received by *x*% of the volume; HI, homogeneity index; FIF, field-in-field technique; ABP, automated breast planning. The prescribed 90% dose volume was created from isodose line in each FIF and ABP original isocenter plans. Data are presented as the mean ± standard deviation [range]. The *p* values are from the paired *t* test.

### Dose for organ at risk

**Figure 2:**
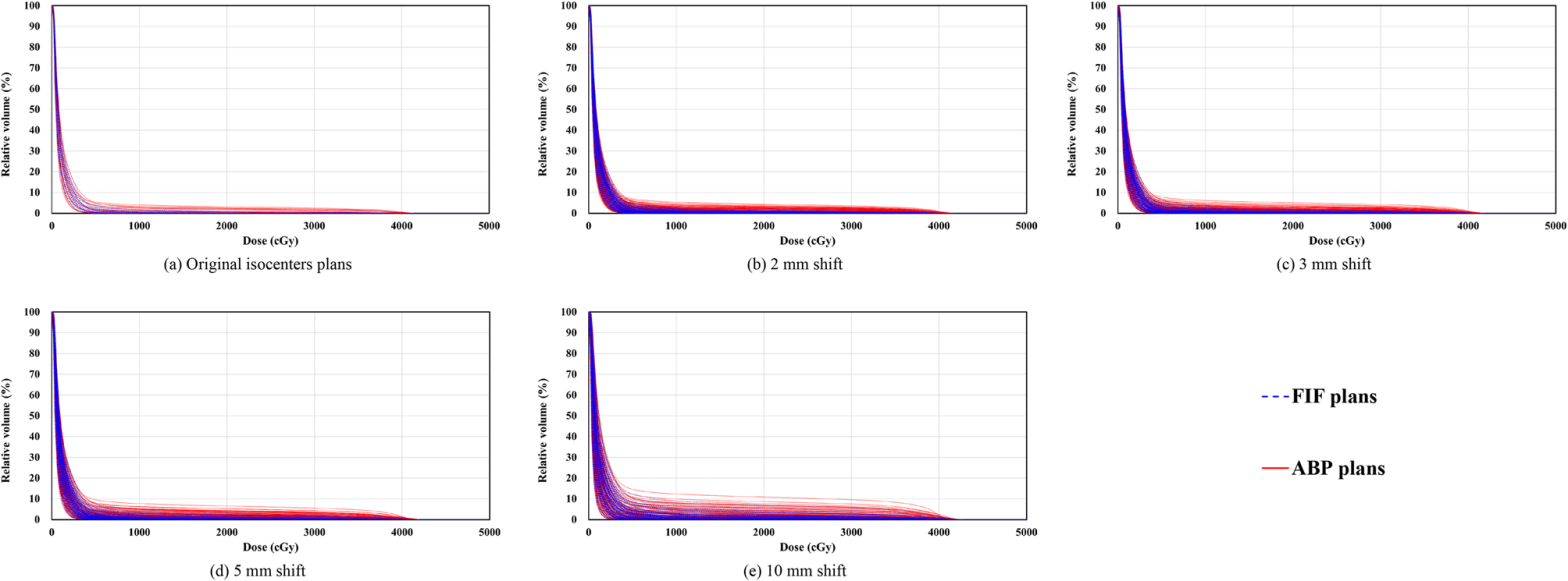
Comparison of dose-volume histograms of the heart among FIF plans and ABP plans. FIF, field-in-field technique; ABP, automated breast planning.

**Figure 3:**
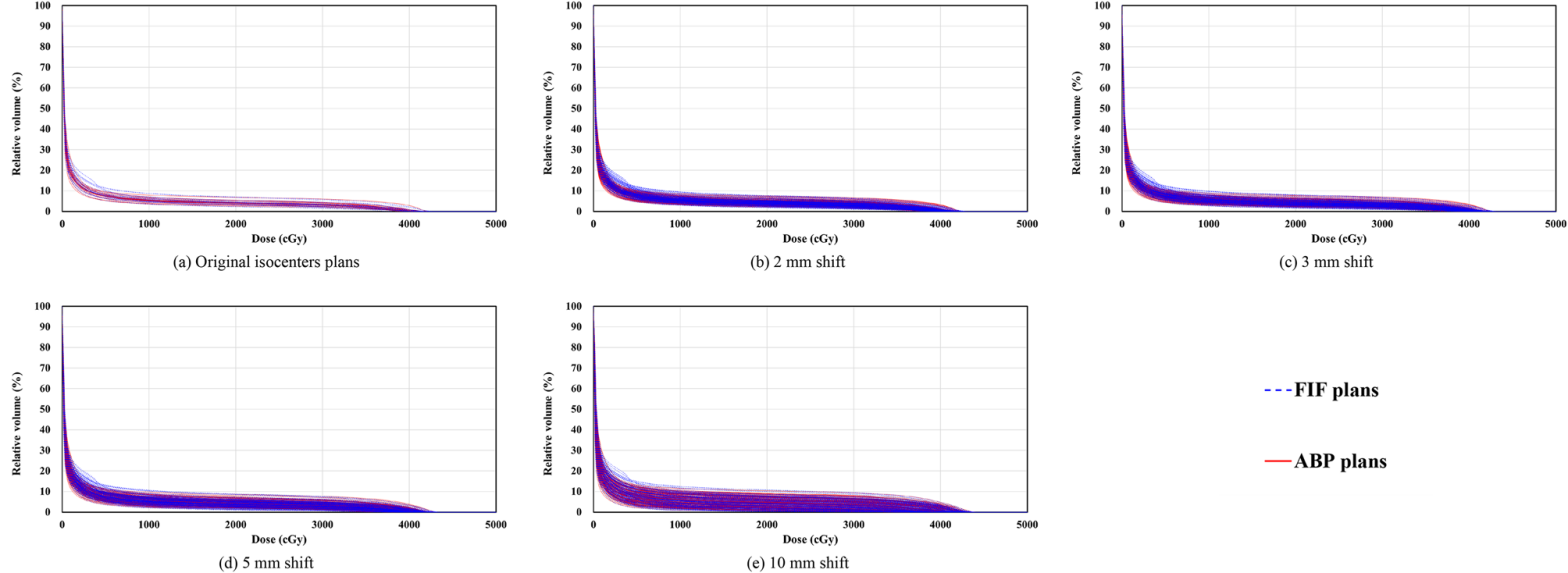
Comparison of dose-volume histograms of bilateral lungs among FIF plans and ABP plans. FIF, field-in-field technique; ABP, automated breast planning.

Table 2 presents the analysis of dose-volume data of the heart and bilateral lungs. The *D*_mean_ value for the heart of the original ABP plans (128.7 ± 53.9 cGy) was significantly higher (*p* = 0.006) than the value in the original FIF plans (103.0 ± 26.9 cGy). In all isocenter-shifted scenarios (2, 3, 5, and 10 mm), the *D*_mean_ values for the heart of ABP plans (129.7 ± 55.6 cGy, 130.9 ± 59.1 cGy, 134.9 ± 69.2 cGy, 152.2 ± 106.8 cGy) were significantly higher (*p* < 0.001) than the values in FIF plans (103.8 ± 28.8 cGy, 104.8 ± 31.8 cGy, 108.0 ± 40.2 cGy, 123.3 ± 71.9 cGy). The differences between FIF plans and ABP plans for the *V*_20 Gy_ value of bilateral lungs were not significant in the original isocenters (FIF: 4.2 ± 1.3%; ABP: 4.2 ± 1.0%; *p* = 0.989). In all isocenter-shifted scenarios (2, 3, 5, and 10 mm), the *V*_20 Gy_ values for bilateral lungs of ABP plans (4.2 ± 1.1%, 4.2 ± 1.2%, 4.2 ± 1.5%, 4.2 ± 2.5%) showed no significant differences (*p* = 0.966, 0.959, 0.948, 0.984) compared with FIF plans (4.2 ± 1.4%, 4.2 ± 1.5%, 4.2 ± 1.8%, 4.2 ± 2.7%). Figures 2 and 3 show the dose-volume histograms of heart and bilateral lungs among FIF and ABP plans. In the 10-mm shifted scenario, several ABP plans had a larger high dose volume of the heart (e.g., > 25 Gy) than FIF plans. No differences were observed for the bilateral lungs dose between FIF and ABP plans in all situations.

**Table 2:** Analysis of dose-volume data of the heart and bilateral lungs.

Isocenter shift (mm)	Heart/*D*_mean_ (cGy)		*p*	Bilateral lungs/*V*_20 Gy_ (%)		*p*
	FIF plans	ABP plans		FIF plans	ABP plans	
0	103.0 ± 26.9 [68.0–155.1]	128.7 ± 53.9 [64.5–228.9]	0.006	4.2 ± 1.3 [2.3–7.1]	4.2 ± 1.0 [2.6–6.9]	0.989
2	103.8 ± 28.8 [62.6–187.1]	129.7 ± 55.6 [58.4–281.4]	< 0.001	4.2 ± 1.4 [1.8–7.8]	4.2 ± 1.1 [2.1–7.6]	0.966
3	104.8 ± 31.8 [60.1–209.0]	130.9 ± 59.1 [55.9–310.3]	< 0.001	4.2 ± 1.5 [1.5–8.1]	4.2 ± 1.2 [1.8–7.9]	0.959
5	108.0 ± 40.2 [54.4–259.0]	134.9 ± 69.2 [51.2–372.6]	< 0.001	4.2 ± 1.8 [1.0–8.9]	4.2 ± 1.5 [1.4–8.6]	0.948
10	123.3 ± 71.9 [41.3–415.6]	152.2 ± 106.8 [40.9–549.9]	< 0.001	4.3 ± 2.7 [0.3–10.7]	4.3 ± 2.5 [0.4–10.4]	0.984

*D*_mean_, mean dose; *V*_*x* Gy_, percentage volume receiving dose greater than *x* Gy; FIF, field-in-field technique; ABP, automated breast planning. Data are presented as the mean ± standard deviation [range]. The *p* values are from the paired *t* test.

## Discussion

This study confirmed that ABP plans were more homogeneous for the target dose than FIF plans in the situations of shifting the beam isocenters within 5 mm. The finding of superiority of ABP plans as hybrid IMRT was similar to previous study findings, although the hybrid plan was generated fully automatically^16,17^. In all situations, the minimum dose represented by *D*_98%_ values of the target region was equal between FIF and ABP plans, and the maximum dose represented by *D*_2%_ values of ABP plans was lower compared with FIF plans. Accordingly, ABP plans had fewer hot spots than FIF plans, although the prescribed dose for the target was ensured. Even if the isocenter shifting was performed with 10 mm, the *HI* value of ABP plans was not significantly poorer compared with FIF plans. Although previous studies investigated the effect of setup uncertainties for the breast IMRT technique, the simulations for setup errors were performed with limited directions and amplitudes^14,17^. The results of this study indicated that ABP plans are not inferior to FIF plans in general clinical situations and that APB plans are superior to FIF plans in most cases for target coverage and homogeneity.

The robustness of ABP plans was caused by the clinically efficient setting of the automated planning algorithms for generating the beams. Table 3 presents the comparison of beam parameters for FIF and ABP plans. The ABP plans had a significantly larger (*p* < 0.001) number of segments for providing a homogeneous target dose by intensity modulation of the beams. However, the differences between FIF and ABP plans for MUs of open segments were not significant (FIF: 143.9 ± 7.9 MU, ABP: 142.3 ± 5.1 MU; *p* = 0.324). The balanced setting of ABP algorithms between the intensity-modulated fields and open fields led to superiority in terms of robustness against geometric uncertainties compared with FIF plans. All ABP plans were automatically generated as hybrid IMRT plans with open fields for the breast flash. Generally, the open fields for the breast flash were not considered in optimization modules for inverse planning or required some cumbersome procedures for the planning. The ABP software can generate a plan in approximately 5 min; therefore, it may be a suitable tool for several institutions where hybrid IMRT plans cannot be used for their patients due to the cost of planning time^11,13^.

**Table 3:** Comparison of beam parameter.

Parameter	*n*	FIF plans	ABP plans	*p*
Number of segments/plan	20	4.5 ± 1.2 [3.0–9.0]	9.9 ± 1.4 [7.0–13.0]	< 0.001
Total MUs/plan	20	325.0 ± 14.6 [303.0–357.0]	361.4 ± 12.6 [334.5–381.9]	< 0.001
MUs of open segment/beam	40	143.9 ± 7.9 [126.7–158.4]	142.3 ± 5.1 [132.9–153.8]	0.324
Weight of open segment/beam (%)	40	88.8 ± 5.8 [73.8–100.0]	78.8 ± 3.6 [71.5–85.8]	< 0.001

FIF, Field-in-field technique; ABP, automated breast planning; MUs, monitor units. Data are presented as the mean ± standard deviation [range]. The *p* values are from the paired *t* test.

The dose for the organ at risk was evaluated for the heart and bilateral lungs. The heart dose in the original ABP plans was significantly higher compared with that for the original FIF plans. This result was the same as in the previous study, and we consider that the difference of the heart dose was caused by aggressive heart shielding for FIF plans in our institution^13^. The tendencies for the heart dose between FIF and ABP plans were observed in all scenarios of shifting beam isocenters. The average of *D*_mean_ value for the heart in FIF and ABP plans was clinically acceptable in all scenarios. However, high dose volume of the heart was increased in several 10-mm shifted ABP plans. Although the 10-mm systematically shifted scenario for all fractionations is unlikely in common clinical situations, in the case of trade-off between the target coverage and the risk of cardiac dose escalation, the combination of hybrid IMRT and the deep inspiration breath-hold technique is considered the optimal setting for patients^22–24^.

This study has several limitations. First, this study demonstrated geometric uncertainties using beam isocenter shifting, for which it was hypothesized that the same setup errors occur systematically across all fractions in a treatment course. The effects of random shift changes during a treatment course with different numbers of fraction were not simulated. Additionally, the dosimetric impact of the interplay effect and organ deformation due to respiratory motion was not validated^25,26^. A previous study reported that no special consideration is required for breast IMRT with typical fractionation^27^. However, it may be safe to consider using breath-hold and/or the image-guided technique for the treatment of a small number of fractions such as an ultra-hypofractionations^28^.

In conclusion, the hybrid IMRT plans generated by commercially available ABP software were superior to forward-planned FIF plans in terms of robustness against geometric uncertainties. The ABP software has the potential to provide high-quality and robust treatment for a large number of breast cancer patients without increasing planning time.

## Data Availability

The datasets used and analyzed during the current study are available upon reasonable request. Please contact the corresponding author for data requests.
